# Supplementary material for: High prevalence of off-label and unlicensed paediatric prescribing in a hospital in Indonesia during the period Aug.—Oct. 2014
Source: PLoS One. 2020 Jan 14;15(1):e0227687. doi: 10.1371/journal.pone.0227687 (PMC6959587; doi:10.1371/journal.pone.0227687)
Supplement: S1 Table — (DOCX) [file pone.0227687.s001.docx]

**Supplement 1**

**All drugs with off-label and unlicensed status**

| **DRUGS** | **PRESCRIBED ON LABEL** | | **PRESCRIBED OFF LABEL** | | **UNLICENSED** | | **Total** | |
| --- | --- | --- | --- | --- | --- | --- | --- | --- |
|  | **n** | **%** | **n** | **%** | **n** | **%** | **n** | % |
| Paracetamol | 59 | 24.0 | 187 | 76.0 |  |  | 246 | 12.5 |
| Ranitidine | 1 | 0.4 | 229 | 99.6 |  |  | 230 | 11.7 |
| Cefotaxime | 26 | 13.9 | 161 | 86.1 |  |  | 187 | 9.5 |
| Ondansetron |  |  | 174 | 100.0 |  |  | 174 | 8.9 |
| Zinc Sulfate | 97 | 72.4 | 37 | 27.6 |  |  | 134 | 6.8 |
| Liprolac | 88 | 73.9 | 31 | 26.1 |  |  | 119 | 6.1 |
| Cefixime | 12 | 10.4 | 88 | 76.5 | 15 | 13.0 | 115 | 5.9 |
| Gentamicin | 9 | 8.7 | 95 | 91.3 |  |  | 104 | 5.3 |
| Ceftriaxone | 16 | 23.2 | 53 | 76.8 |  |  | 69 | 3.5 |
| Artesunate |  |  | 57 | 100.0 |  |  | 57 | 2.9 |
| Primaquine | 4 | 10.8 | 15 | 40.5 | 18 | 48.6 | 37 | 1.9 |
| Xanvit | 24 | 64.9 | 13 | 35.1 |  |  | 37 | 1.9 |
| Dexamethasone |  |  | 35 | 100.0 |  |  | 35 | 1.8 |
| Darplex |  |  |  |  | 30 | 100.0 | 30 | 1.5 |
| Cough powder 14 |  |  |  |  | 29 | 100.0 | 29 | 1.5 |
| Dialac | 7 | 30.4 | 16 | 69.6 |  |  | 23 | 1.2 |
| Ambroxol | 4 | 20.0 | 16 | 80.0 |  |  | 20 | 1.0 |
| Metronidazole | 2 | 12.5 | 12 | 75.0 | 2 | 12.5 | 16 | 0.8 |
| L- Bio |  |  | 13 | 100.0 |  |  | 13 | 0.7 |
| Vitamin A | 9 | 69.2 | 4 | 30.8 |  |  | 13 | 0.7 |
| San-b-plex | 7 | 58.3 | 5 | 41.7 |  |  | 12 | 0.6 |
| Nebuliser 1 |  |  | 11 | 100.0 |  |  | 11 | 0.6 |
| Nebuliser 2 |  |  | 10 | 100.0 |  |  | 10 | 0.5 |
| Fe elemental |  |  | 3 | 33.3 | 6 | 66.7 | 9 | 0.5 |
| Cotrimoxazole 240 | 4 | 50.0 | 4 | 50.0 |  |  | 8 | 0.4 |
| Methylprednisolone | 1 | 12.5 | 7 | 87.5 |  |  | 8 | 0.4 |
| Nystatin drops | 2 | 25.0 | 6 | 75.0 |  |  | 8 | 0.4 |
| Antasida DOEN | 2 | 28.6 | 5 | 71.4 |  |  | 7 | 0.4 |
| Colistin Sulfate |  |  | 3 | 42.9 | 4 | 57.1 | 7 | 0.4 |
| Nebuliser 5 |  |  | 7 | 100.0 |  |  | 7 | 0.4 |
| Phenobarbital | 1 | 14.3 |  |  | 6 | 85.7 | 7 | 0.4 |
| Sucralfate |  |  | 2 | 28.6 | 5 | 71.4 | 7 | 0.4 |
| Aminophylline | 1 | 16.7 | 5 | 83.3 |  |  | 6 | 0.3 |
| Cough powder 3 |  |  |  |  | 6 | 100.0 | 6 | 0.3 |
| Fluconazole |  |  | 2 | 33.3 | 4 | 66.7 | 6 | 0.3 |
| Imboost force |  |  | 6 | 100.0 |  |  | 6 | 0.3 |
| Omeprazole |  |  | 6 | 100.0 |  |  | 6 | 0.3 |
| Phenytoin |  |  | 4 | 66.7 | 2 | 33.3 | 6 | 0.3 |
| Piracetam |  |  | 6 | 100.0 |  |  | 6 | 0.3 |
| Diazepam | 5 | 100.0 |  |  |  |  | 5 | 0.3 |
| Domperidone |  |  | 5 | 100.0 |  |  | 5 | 0.3 |
| Erdosteine | 1 | 20.0 | 4 | 80.0 |  |  | 5 | 0.3 |
| Ferrous Sulfate |  |  | 5 | 100.0 |  |  | 5 | 0.3 |
| Folic acid |  |  | 5 | 100.0 |  |  | 5 | 0.3 |
| Pirantel pamoate | 4 | 80.0 |  |  | 1 | 20.0 | 5 | 0.3 |
| Amikacin |  |  | 4 | 100.0 |  |  | 4 | 0.2 |
| Cefadroxil |  |  | 4 | 100.0 |  |  | 4 | 0.2 |
| Ferokid syrup |  |  | 4 | 100.0 |  |  | 4 | 0.2 |
| OBH Comby anak |  |  | 4 | 100.0 |  |  | 4 | 0.2 |
| Chloramphenicol | 3 | 100.0 |  |  |  |  | 3 | 0.2 |
| Cough Powder 6 |  |  |  |  | 3 | 100.0 | 3 | 0.2 |
| Ketorolac |  |  | 3 | 100.0 |  |  | 3 | 0.2 |
| Meropenem |  |  | 3 | 100.0 |  |  | 3 | 0.2 |
| OAT 1 |  |  |  |  | 3 | 100.0 | 3 | 0.2 |
| OAT 3 | 1 | 33.3 |  |  | 2 | 66.7 | 3 | 0.2 |
| Prednisone |  |  | 3 | 100.0 |  |  | 3 | 0.2 |
| Cotrimoxazole | 1 | 50.0 | 1 | 50.0 |  |  | 2 | 0.1 |
| Cough powder 9 |  |  |  |  | 2 | 100.0 | 2 | 0.1 |
| Furosemide |  |  | 2 | 100.0 |  |  | 2 | 0.1 |
| Nebuliser 4 | 1 | 50.0 | 1 | 50.0 |  |  | 2 | 0.1 |
| Nebuliser 8 |  |  | 2 | 100.0 |  |  | 2 | 0.1 |
| Nebuliser 9 |  |  | 2 | 100.0 |  |  | 2 | 0.1 |
| Neurobion | 2 | 100.0 |  |  |  |  | 2 | 0.1 |
| Pantoprazole |  |  | 2 | 100.0 |  |  | 2 | 0.1 |
| Rhinos |  |  | 2 | 100.0 |  |  | 2 | 0.1 |
| Sanmag |  |  |  |  | 2 | 100.0 | 2 | 0.1 |
| Tramadol |  |  | 2 | 100.0 |  |  | 2 | 0.1 |
| Vitamin B Complex | 2 | 100.0 |  |  |  |  | 2 | 0.1 |
| Amoxicillin |  |  | 1 | 100.0 |  |  | 1 | 0.1 |
| Ampicillin |  |  | 1 | 100.0 |  |  | 1 | 0.1 |
| Bactrim | 1 | 100.0 |  |  |  |  | 1 | 0.1 |
| Cetirizine | 1 | 100.0 |  |  |  |  | 1 | 0.1 |
| Ciprofloxacin |  |  | 1 | 100.0 |  |  | 1 | 0.1 |
| Colme ear drop | 1 | 100.0 |  |  |  |  | 1 | 0.1 |
| Cough powder 1 |  |  |  |  | 1 | 100.0 | 1 | 0.1 |
| Cough powder 10 |  |  |  |  | 1 | 100.0 | 1 | 0.1 |
| Cough powder 11 |  |  |  |  | 1 | 100.0 | 1 | 0.1 |
| Cough powder 12 |  |  |  |  | 1 | 100.0 | 1 | 0.1 |
| Cough powder 13 |  |  |  |  | 1 | 100.0 | 1 | 0.1 |
| Cough powder 2 |  |  |  |  | 1 | 100.0 | 1 | 0.1 |
| Cough powder 4 |  |  |  |  | 1 | 100.0 | 1 | 0.1 |
| Cough powder 5 |  |  |  |  | 1 | 100.0 | 1 | 0.1 |
| Cough powder 7 |  |  |  |  | 1 | 100.0 | 1 | 0.1 |
| Cough powder 8 |  |  |  |  | 1 | 100.0 | 1 | 0.1 |
| Elkana syrup |  |  | 1 | 100.0 |  |  | 1 | 0.1 |
| Erythromycin | 1 | 100.0 |  |  |  |  | 1 | 0.1 |
| Ethambutol |  |  | 1 | 100.0 |  |  | 1 | 0.1 |
| FG Troches | 1 | 100.0 |  |  |  |  | 1 | 0.1 |
| Haloperidol |  |  | 1 | 100.0 |  |  | 1 | 0.1 |
| Hepamax |  |  |  |  | 1 | 100.0 | 1 | 0.1 |
| Hydrogen peroxide | 1 | 100.0 |  |  |  |  | 1 | 0.1 |
| Ibuprofen |  |  |  |  | 1 | 100.0 | 1 | 0.1 |
| Interlac drops | 1 | 100.0 |  |  |  |  | 1 | 0.1 |
| Ipratropium Bromide |  |  | 1 | 100.0 |  |  | 1 | 0.1 |
| Levofloxacin |  |  | 1 | 100.0 |  |  | 1 | 0.1 |
| Mebendazole |  |  |  |  | 1 | 100.0 | 1 | 0.1 |
| Nebuliser 3 |  |  | 1 | 100.0 |  |  | 1 | 0.1 |
| Nebuliser 6 |  |  | 1 | 100.0 |  |  | 1 | 0.1 |
| Nifuroxazide |  |  | 1 | 100.0 |  |  | 1 | 0.1 |
| Nystatin |  |  | 1 | 100.0 |  |  | 1 | 0.1 |
| OAT 2 |  |  | 1 | 100.0 |  |  | 1 | 0.1 |
| OAT 4 |  |  |  |  | 1 | 100.0 | 1 | 0.1 |
| Pseudoephedrine HCl |  |  | 1 | 100.0 |  |  | 1 | 0.1 |
| Salbutamol |  |  | 1 | 100.0 |  |  | 1 | 0.1 |
| Sistenol | 1 | 100.0 |  |  |  |  | 1 | 0.1 |
| Thiamphenicol |  |  | 1 | 100.0 |  |  | 1 | 0.1 |
| Tranexamic acid |  |  | 1 | 100.0 |  |  | 1 | 0.1 |
|  |  |  |  |  |  |  |  |  |
| **Total** | **404** | **20.6** | **1403** | **71.5** | **154** | **7.9** | **1961** | **100** |
