# Supplementary material for: High prevalence of off-label and unlicensed paediatric prescribing in a hospital in Indonesia during the period Aug.—Oct. 2014
Source: PLoS One. 2020 Jan 14;15(1):e0227687. doi: 10.1371/journal.pone.0227687 (PMC6959587; doi:10.1371/journal.pone.0227687)
Supplement: S2 Table — (DOCX) [file pone.0227687.s002.docx]

**Supplement 2**

**Reasons, or combination of reasons, for off-label prescribing**

| **Drug** | **Reason for off-label classification** | | | | | | | |
| --- | --- | --- | --- | --- | --- | --- | --- | --- |
|  | **Age/ weight** | **Indication** | **Dose/ frequency** | **CI** | **CI for age** | **CI for diagnosis** | **Special Precautions** | **Not Recommended** |
| Ambroxol (n = 16) |  |  |  |  |  |  |  |  |
| Aminophylline (n = 5) |  |  |  |  |  |  |  |  |
| Amikacin (n = 4) |  |  |  |  |  |  |  |  |
| Amoxicillin (n = 1) |  |  |  |  |  |  |  |  |
| Ampicillin (n = 1) |  |  |  |  |  |  |  |  |
| Antasida DOEN (n = 1) |  |  |  |  |  |  |  |  |
| Antasida DOEN (n = 1) |  |  |  |  |  |  |  |  |
| Antasida DOEN (n = 1) |  |  |  |  |  |  |  |  |
| Antasida DOEN (n = 1) |  |  |  |  |  |  |  |  |
| Antasida DOEN (n = 1) |  |  |  |  |  |  |  |  |
| Artesunate (n = 1) |  |  |  |  |  |  |  |  |
| Artesunate (n = 1) |  |  |  |  |  |  |  |  |
| Artesunate (n = 17) |  |  |  |  |  |  |  |  |
| Artesunate (n = 38) |  |  |  |  |  |  |  |  |
| Cefadroxil (n = 2) |  |  |  |  |  |  |  |  |
| Cefadroxil (n = 2) |  |  |  |  |  |  |  |  |
| Cefixime (n = 5) |  |  |  |  |  |  |  |  |
| Cefixime (n = 6) |  |  |  |  |  |  |  |  |
| Cefixime (n = 9) |  |  |  |  |  |  |  |  |
| Cefixime (n = 27) |  |  |  |  |  |  |  |  |
| Cefixime (n = 41) |  |  |  |  |  |  |  |  |
| Cefotaxime (n = 1) |  |  |  |  |  |  |  |  |
| Cefotaxime (n = 4) |  |  |  |  |  |  |  |  |
| Cefotaxime (n = 156) |  |  |  |  |  |  |  |  |
| Ceftriaxone (n = 2) |  |  |  |  |  |  |  |  |
| Ceftriaxone (n = 51) |  |  |  |  |  |  |  |  |
| Ciprofloxacin (n = 1) |  |  |  |  |  |  |  |  |
| Colistin sulfate (n = 3) |  |  |  |  |  |  |  |  |
| Cotrimoxazole (n = 5) |  |  |  |  |  |  |  |  |
| Dexamethasone (n = 1) |  |  |  |  |  |  |  |  |
| Dexamethasone (n = 1) |  |  |  |  |  |  |  |  |
| Dexamethasone (n = 6) |  |  |  |  |  |  |  |  |
| Dexamethasone (n = 27) |  |  |  |  |  |  |  |  |
| Dialac (n = 1) |  |  |  |  |  |  |  |  |
| Dialac (n = 15) |  |  |  |  |  |  |  |  |
| Domperidone (n = 1) |  |  |  |  |  |  |  |  |
| Domperidone (n = 4) |  |  |  |  |  |  |  |  |
| Elkana syrup (n = 1) |  |  |  |  |  |  |  |  |
| Erdosteine (n = 1) |  |  |  |  |  |  |  |  |
| Erdosteine (n = 3) |  |  |  |  |  |  |  |  |
| Ethambutol (n = 1) |  |  |  |  |  |  |  |  |
| Fe elemental (n = 1) |  |  |  |  |  |  |  |  |
| Fe elemental (n = 2) |  |  |  |  |  |  |  |  |
| Ferokid syrup (n = 1) |  |  |  |  |  |  |  |  |
| Ferokid syrup (n = 3) |  |  |  |  |  |  |  |  |
| Ferrous sulfate (n = 4) |  |  |  |  |  |  |  |  |
| Fluconazole (n = 2) |  |  |  |  |  |  |  |  |
| Folic acid (n = 5) |  |  |  |  |  |  |  |  |
| Furosemide (n = 2) |  |  |  |  |  |  |  |  |
| Gentamicin (n = 16) |  |  |  |  |  |  |  |  |
| Gentamicin (n = 23) |  |  |  |  |  |  |  |  |
| Gentamicin (n = 56) |  |  |  |  |  |  |  |  |
| Haloperidol (n = 1) |  |  |  |  |  |  |  |  |
| Imboost Force (n = 6) |  |  |  |  |  |  |  |  |
| Ipratropium bromide (n = 1) |  |  |  |  |  |  |  |  |
| Ketorolac (n = 3) |  |  |  |  |  |  |  |  |
| L-Bio (n = 2) |  |  |  |  |  |  |  |  |
| L-Bio (n = 11) |  |  |  |  |  |  |  |  |
| Levofloxacin (n = 1) |  |  |  |  |  |  |  |  |
| Liprolac (n = 31) |  |  |  |  |  |  |  |  |
| Meropenem (n = 1) |  |  |  |  |  |  |  |  |
| Meropenem (n = 1) |  |  |  |  |  |  |  |  |
| Meropenem (n = 1) |  |  |  |  |  |  |  |  |
| Methylprednisolone (n = 1) |  |  |  |  |  |  |  |  |
| Methylprednisolone (n = 2) |  |  |  |  |  |  |  |  |
| Methylprednisolone (n = 1) |  |  |  |  |  |  |  |  |
| Methylprednisolone (n = 3) |  |  |  |  |  |  |  |  |
| Metronidazole (n = 4) |  |  |  |  |  |  |  |  |
| Metronidazole (n = 8) |  |  |  |  |  |  |  |  |
| Nebuliser 1 (n = 1) |  |  |  |  |  |  |  |  |
| Nebuliser 1 (n = 10) |  |  |  |  |  |  |  |  |
| Nebuliser 2 (n = 1) |  |  |  |  |  |  |  |  |
| Nebuliser 2 (n = 9) |  |  |  |  |  |  |  |  |
| Nebuliser 4 (n = 1) |  |  |  |  |  |  |  |  |
| Nebuliser 5 (n = 7) |  |  |  |  |  |  |  |  |
| Nebuliser 6 (n = 1) |  |  |  |  |  |  |  |  |
| Nebuliser 8 (n = 1) |  |  |  |  |  |  |  |  |
| Nebuliser 8 (n = 1) |  |  |  |  |  |  |  |  |
| Nebuliser 9 (n = 2) |  |  |  |  |  |  |  |  |
| Nitrofuroxazide (n = 1) |  |  |  |  |  |  |  |  |
| Nystatin drops (n = 7) |  |  |  |  |  |  |  |  |
| OAT 2 (n = 1) |  |  |  |  |  |  |  |  |
| OBH Combi anak (n = 1) |  |  |  |  |  |  |  |  |
| OBH Combi anak (n = 3) |  |  |  |  |  |  |  |  |
| Omeprazole (n = 6) |  |  |  |  |  |  |  |  |
| Ondansetron (n = 1) |  |  |  |  |  |  |  |  |
| Ondansetron (n = 1) |  |  |  |  |  |  |  |  |
| Ondansetron (n = 45) |  |  |  |  |  |  |  |  |
| Ondansetron (n = 127) |  |  |  |  |  |  |  |  |
| Pantoprazole (n = 1) |  |  |  |  |  |  |  |  |
| Pantoprazole (n = 1) |  |  |  |  |  |  |  |  |
| Paracetamol (n = 39) |  |  |  |  |  |  |  |  |
| Paracetamol (n = 148) |  |  |  |  |  |  |  |  |
| Phenytoin (n = 1) |  |  |  |  |  |  |  |  |
| Phenytoin (n = 1) |  |  |  |  |  |  |  |  |
| Phenytoin (n = 2) |  |  |  |  |  |  |  |  |
| Piracetam (n = 6) |  |  |  |  |  |  |  |  |
| Prednisone (n = 1) |  |  |  |  |  |  |  |  |
| Prednisone (n = 2) |  |  |  |  |  |  |  |  |
| Primaquine (n = 1) |  |  |  |  |  |  |  |  |
| Primaquine (n = 14) |  |  |  |  |  |  |  |  |
| Pseudoephedrine (n = 1) |  |  |  |  |  |  |  |  |
| Ranitidine (n = 1) |  |  |  |  |  |  |  |  |
| Ranitidine (n = 1) |  |  |  |  |  |  |  |  |
| Ranitidine (n = 1) |  |  |  |  |  |  |  |  |
| Ranitidine (n = 1) |  |  |  |  |  |  |  |  |
| Ranitidine (n = 6) |  |  |  |  |  |  |  |  |
| Ranitidine (n = 219) |  |  |  |  |  |  |  |  |
| Rhinos (n = 2) |  |  |  |  |  |  |  |  |
| Salbutamol (n = 1) |  |  |  |  |  |  |  |  |
| San-b-plex (n = 5) |  |  |  |  |  |  |  |  |
| Sucralfate (n = 2) |  |  |  |  |  |  |  |  |
| Thiamphenicol (n = 1) |  |  |  |  |  |  |  |  |
| Tramadol (n = 1) |  |  |  |  |  |  |  |  |
| Tramadol (n = 1) |  |  |  |  |  |  |  |  |
| Tranexamic acid (n = 1) |  |  |  |  |  |  |  |  |
| Vitamin A (n = 4) |  |  |  |  |  |  |  |  |
| Xanvit (n = 13) |  |  |  |  |  |  |  |  |
| Zinc sulfate (n = 2) |  |  |  |  |  |  |  |  |
| Zinc sulfate (n = 35) |  |  |  |  |  |  |  |  |
