# Supplementary material for: High prevalence of off-label and unlicensed paediatric prescribing in a hospital in Indonesia during the period Aug.—Oct. 2014
Source: PLoS One. 2020 Jan 14;15(1):e0227687. doi: 10.1371/journal.pone.0227687 (PMC6959587; doi:10.1371/journal.pone.0227687)
Supplement: Attachment — Submitted filename: Response to Reviewers 041219.docx [file pone.0227687.s003.docx]

**Response to Reviewer’s Comments**

**High prevalence of off-label and unlicensed paediatric prescribing in a hospital in Indonesia during the period Aug. – Oct. 2014**

We would like to thank the reviewers for their comments. We have considered all of the comments carefully and made several modifications to the manuscript.

| **Comment** | **Authors Responses** |
| --- | --- |
| Reviewer #1: | |
| 1. The authors state that with the small number of cases in this table from the adolescents, it was found more appropriate to quote the p-value obtained from Fisher's exact test. But in the section of statistical analysis of the revised manuscript, they didn't mention about Fisher's exact test. | Thank you for your comment. A statement addressing this oversight has now been added. |
| 2. Did the authors consider to exclude six adolescents from GEE model and Fisher's exact test / Chi-square test? Because there were only six adolescent patients in the data, the sample size of the adolescents was small. The situation of six adolescent patients could be discussed separately. | We developed the GEE model using all the data (including the adolescents), but then re-ran the model after excluding the records belonging to these 6 patients. We found that the two models agreed closely, and that the statistical significance of the independent variables identified using the model based on all the records was very similar to those obtained after excluding the adolescents. On this basis, we felt it was justified to include the 57 off-label drug records belonging to these 6 adolescent patients. |
| 3. The items in Table 2 are confused. Some drugs have two frequencies (e.g., paracetamol, ondansetron, ...). The cumulative frequency of age/weight, indication, dose/frequency, and special precautions for ranitidine is 243, which was not equal to the frequency of ranitidine in Table 2. It might be that the frequency of ranitidine in Table 2 was counted as combination of reasons, but the frequency in Table 3 was not. Even so, it's easy to confuse. Information about Table 2 might be addressed in the context or the footnote, but not in the title of Table 2. | We have modified Table 2 to include all of the ten most commonly prescribed drugs in Table 1. For each drug, the reason, or combination of reasons, for off-label prescribing, is shown in Table 2.  Further, we have added a line to the first column to show the number of patients who were taking each drug (in addition to the number of drug records on which each line of the table was based). This may clarify the situation.  We reviewed Table 3, and found that there was an error in the numbers. We consider that the expansion of Table 2 now includes all the information that we wanted to describe in Table 3, and therefore we think it is best to delete Table 3. It was included only as an example, but we thank the reviewer for pointing out the inconsistency in the numbers. |
| Reviewer #2: | |
| The manuscript has been largely improved by addressing most of the issues raised from previous review. But there is still one issue to be solved, the title does not fit the context. They are suggested to append 'during the period Aug.-Oct. 2014' to the title of manuscript to reflect the context and avoid the over-claiming issue. | Many thanks for your suggestion. The title has been changed to:  ‘High prevalence of off-label and unlicensed paediatric prescribing in a hospital in Indonesia during the period Aug. – Oct. 2014’ |
| Reviewer #3: | |
| The reason is still unknown why the size of study population is only 200 randomly selected patients rather than include all 307 patients? Although 200 is enough to provides a sufficient sample size for regression and related analysis to be performed, how about all 307 patients? Please show the results of non selected dataset. | Under the ethical principles related to the conduct of research, it is unethical to collect additional data than is necessary to demonstrate the objectives which were being pursued, as the data is private and confidential information. In this case, a list of a random sample of 200 medical records (from a population of 307 medical records) was prepared before patient medical records were accessed. Data was only collected from the 200 patient medical records which were randomly selected. As this was a random sample, that sample should be representative of the 307 that constituted the sampling frame. Further, a statistician was consulted to ensure the random sample was sufficient for the purpose of the analysis performed. |
